# Grain yield loss and seed nutritional quality alteration in faba bean (*Vicia faba* L.) caused by the stem borer *Lixus algirus* L. (Coleoptera: Curculionidae)

**DOI:** 10.3389/finsc.2025.1666457

**Published:** 2025-10-30

**Authors:** Mohamed Ouaarous, Hasnae Choukri, Asma Tika, Moez Amri, Adil Baouchi, Chaimae Ramdani, Nezha Ait Taadaouit, Rachid Boulamtat, Issam Meftah Kadmiri, Abderrahim Aasfar, Mansour Sobeh, BadrEddine Drissi, Dina Zanbot, Yaya Sane, Abdelhalim Mesfioui, Mustapha El Bouhssini, Karim El Fakhouri

**Affiliations:** ^1^ AgroBioSciences Program, College of Agriculture and Environmental Sciences, Mohammed VI Polytechnic University, Ben Guerir, Morocco; ^2^ Laboratory of Biology and Health, Department of Biology, Faculty of Science, Ibn-Tofail University, Kenitra, Morocco; ^3^ Entomology Laboratory, International Center for Agricultural Research in the Dry Areas (ICARDA), Rabat, Morocco; ^4^ Plant and Microbial Biotechnology Center, Moroccan Foundation for Advanced Science, Innovation and Research (MAScIR), Mohammed VI Polytechnic University, Ben Guerir, Morocco

**Keywords:** faba bean, Lixus algirus L., yield loss, nutritional quality, protein content, mineral concentration

## Abstract

Faba bean (Vicia faba L.) is one of the most important cool-season legume crops worldwide, particularly in the Mediterranean regions. It plays a crucial role in cereal-based crop rotations and serves as an accessible and cost-effective protein source for both human diets and livestock feed. Despite its significance, faba bean production is heavily impacted by the stem borer Lixus algirus L. (Coleoptera: Curculionoidea), a prominent insect pest in the Mediterranean region. This research aimed to assess the impact of L. algirus on grain yield and seed nutritional profile of a local variety ‘Defes’. The experiment was conducted using insect-proof cages at ICARDA - Marchouch research station during the 2018–2019 and 2019–2020 seasons. The findings revealed that L. algirus infestation caused grain yield losses ranging from 14% to 20%. Larval feeding within plant stems significantly altered seed nutritional composition compared to seeds from non-infested plant, ICP-OES analysis revealed significant declines were observed in magnesium (44%), manganese (38%), calcium (37%), zinc (30%), and iron (27%) concentrations in seeds collected from infested plants. In contrast, an increase in seed protein content and total sugar levels was recorded in infested plant seeds compared to non-infested plants. Similar results were observed for both essential amino acids (such as threonine, isoleucine, leucine, phenylalanine, histidine, lysine, and arginine) and non-essential amino acids (including glutamic acid, tyrosine, and alanine). Multivariate analyses, including PCA and correlation, revealed distinct nutrient and morphological trait patterns between infested and non-infested faba bean samples across both seasons. Collectively, these results show that L. algirus not only reduces grain yield but also reconfigures seed nutritional quality, lowering mineral density despite higher protein and sugars, highlighting the need for integrating host plant resistance for stem borer management and timely IPM to preserve both productivity and food/feed quality.

## 1 Introduction

Faba bean (Vicia faba L.) ranks among the most important legume crops worldwide. It is a major cool-season legume in Morocco with approximately 200,000 hectares of cultivated area. The crop holds a central place in agricultural cropping systems, particularly in rotation with cereals (1–3). Additionally, faba bean is a good source of protein, minerals, vitamins, starch, and provides natural antioxidant compounds for chronic disease prevention and health promotion (4). The overall average dry grain yield of faba beans in Morocco remains 58% lower than the world average during the period 2001–2017 (5). Many stresses are associated with a reduction in faba bean yield, including salinity, rainfall irregularity, drought, and nutrient deficiency (2, 6). Moreover, faba bean is vulnerable to various biotic stress factors such as Orobanche spp., Botrytis infections, viral pathogens, stem nematodes, and insect pests, all of which have significantly reduced its productivity and overall availability (2).

The faba bean stem borer Lixus algirus L. (Coleoptera: Curculionidae) is regarded as a significant biotic threat to faba bean cultivation in several Mediterranean regions, particularly in Morocco (7). Adult L. algirus begin feeding on the leaf edges of faba bean plants from late winter through late spring, showing a preference for the tender upper leaves. This feeding results in characteristic marginal, semi-circular notches and occasionally leaves behind dark fecal spots. In Morocco, a single generation of L. algirus has been observed on faba bean varieties, with an average infestation rate of 75% (8, 9). Most of the damage occurs when larvae feed internally inside the stems, a threat that is particularly critical for young faba bean plants. Such feeding damage disrupts the movement of xylem and phloem. In general, the larval development cycle typically spans around 42 days (8). The liquid nutrients are cut off from the rest of the plant, causing yellowing of leaves and growth-stunting, wilting, and drying of the plant at the vegetative stage (8, 10, 11) and affecting crop growth and yield (12).

Recent advances in the integrated pest management of L. algirus have highlighted the potential of combining host plant resistance and biological control, leading to significant decreases in pest pressure under field conditions. Recent studies showed that planting date did not show a significant effect on L. algirus infestation of faba bean genotypes across locations in Morocco (8). The same authors have reported two genotypes, IG 11561 and IG 72498, showing a high level of resistance to L. algirus, the combination of antibiosis and antixenosis categories are responsible for the resistance. Other studies have reported several natural enemies of L. algirus, including egg parasitoids, an egg predator, a larval parasitoid, and a parasitoid wasp that could have a significant positive impact on the control of L. algirus in faba bean (8, 13–15). Despite the advances of research, faba bean stem borer management remains dependent mainly on chemical insecticides as an effective alternative. Non-chemical control strategies are yet to be developed or widely adopted.

In Morocco, L. algirus is a major biotic constraint to faba bean production, with its stem-boring activity negatively affecting plant performance and seed development. Although the presence of L. algirus in faba bean fields is well documented, precise estimates of the associated yield losses and, more critically, the extent to which infestation alters the nutritional quality of harvested seeds remain poorly quantified and overlooked. These knowledge gaps limit the development of effective pest management strategies and the advancement of tolerant cultivars. The objectives of this study were 1) to quantify yield losses caused by L. algirus infestation under field conditions. 2) to assess the impact of infestation on the nutritional quality of faba bean seeds, and 3) to identify infestation-related patterns and trait relationships using multivariate analysis.

## 2 Materials and methods

### 2.1 Yield loss estimation: insect-proof cages experiment

The experiment was conducted during the 2018–2019 and 2019–2020 cropping seasons at the ICARDA Marchouch Research Station (33°36′33.8″N, 6°42′51.8″W). Weather data, including temperature and rainfall, were recorded at the station.

**Figure 1:**
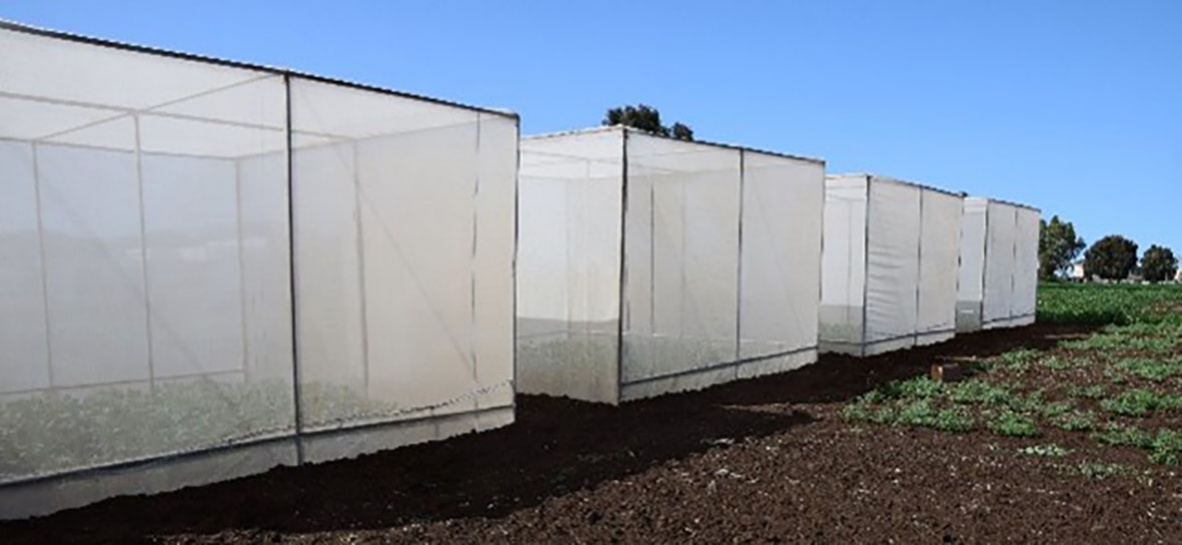
Cage-based field trials to assess yield and quality losses by faba bean stem borer *L. algirus*, Marchouch Station, 2018/19 and 2019/20 cropping seasons.

The local Moroccan faba bean variety ‘Defes’ (Vicia faba var. major) was planted on 9 December 2018 and 16 December 2019 in a randomized complete block design with three infested cages and three uninfested controls. Each cage measured 5 × 5 × 3.6 m (**Figure 1**) and was covered with mosquito netting. Within each cage, six rows (3 m long, 0.6 m spacing) were sown at a seeding rate of 100 kg/ha. Standard agronomic practices were applied, weeds were removed manually, and no insecticides were used. Adult L. algirus were collected in early February from faba bean fields near Rabat (33°56′01″N, 6°69′02″W). Insects were held in mesh-covered trays (230 × 450 × 700 mm) and sexed based on rostrum length (16). Males and females were kept separately and provided faba bean leaves. Adults were released into three cages at the early flowering stage at a density of 1.5 females/m² (17). The remaining cages were kept uninfested as controls. Plant samples were collected monthly. Five plants were taken from cage borders and examined in the laboratory for oviposition, larval or pupal presence, and exit holes using a Motic SMZ-140 stereomicroscope. At harvest, plants from the central four rows of each cage were harvested, and seed yield was converted to kg/ha. Yield loss was calculated as:

$Yield\;loss\;\left(\%\right)=\frac{U-I}{U}$ .


$$Yield\;increased\;\left(\%\right)=\frac{U-I}{I}$$

Where,

U: is the mean yield in uninfested cages (Kg ha^-1^).

I: is the mean yield in infested cages (Kg ha^-1^).

### 2.2 Impact of L. algirus infestation on seed size and nutritional quality

#### 2.2.1 Morphological seed characterization

High-resolution images of faba bean seeds were captured using a Canon CanoScan LiDE 700 F scanner (Canon Inc., Tokyo, Japan). To enhance contrast at the edges of the seeds and reduce reflections and shadows, a matte black box was placed over the scanning area. The images were scanned at 300 dpi without any color modifications or cropping. Image analysis was performed using GrainScan software (Cisaro) (18), which enabled precise measurement of seed geometry, including length, width, perimeter, and area. No additional shape-specific calibrations were applied beyond the default GrainScan settings.

#### 2.2.2 Cooking time

Faba bean cooking time was measured using an automated Mattson Cooker with 25 weighted plungers (80 g, 2 mm tip). Seeds (2 g) were soaked in 50 mL distilled water at 22 ± 2°C for 12 hours. Twenty-five soaked seeds were placed in the cooker, submerged in 1.5 L boiling water, and heated on a 390°C hot plate. Cooking time, defined as the time in minutes required for 80% of seeds to be pierced, was recorded automatically when plungers penetrated and activated a sensor. Each treatment was analyzed in triplicate.

#### 2.2.3 Proximate composition

• Moisture content.

Moisture content in faba bean seeds was assessed using the NIRS DS 2500 model (FOSS, Denmark). Calibration was based on moisture content values obtained using the oven-drying method (105 °C for 24 hours, AOAC 925.10). Partial Least Squares Regression (PLSR) was applied to spectral data for analysis, and moisture content was expressed as a percentage of fresh weight. Calibration performance was evaluated using R²= 0.96 and RMSE.

• Ash content.

Ash content in faba bean seeds was measured using the NIRS DS 2500 model (FOSS, Denmark). Calibration was performed using ash content data obtained through the gravimetric method (ASH in AOAC 942.05). Partial Least Squares Regression (PLSR) was employed to analyze the spectral data. The results were expressed in percentage (%). Calibration accuracy was evaluated using R²= 0.86 and RMSE values.

• Fiber content.

Fiber content in faba bean seeds was analyzed using the NIRS DS 2500 model (FOSS, Denmark). The measurement was based on calibration equations developed for fiber content using the standard AOAC Method 978.10 (for crude fiber determination). The data was processed using Partial Least Squares Regression (PLSR) to correlate with the fiber content. Results were expressed as a percentage of the total sample weight. Calibration accuracy was evaluated based on the coefficient of determination (R²= 0.97) and the Root Mean Square Error of Prediction (RMSEP).

• Starch content.

Starch content in faba bean seeds was determined using the NIRS DS 2500 model (FOSS, Denmark) following standard protocols. The spectral data obtained were processed using Partial Least Squares Regression (PLSR), developed from calibration equations for starch content. The calibration was validated according to the AOAC Method 996.11 (for starch determination). Starch content was expressed as a percentage of the total sample weight. The model’s performance was evaluated by the coefficient of determination (R²= 0.87) and the Root Mean Square Error of Prediction (RMSEP) to ensure accuracy and precision.

• Carbohydrate content.

Total sugars were measured based on Dubois’ method after sulfuric acid digestion (19). Briefly, 0.5 ml of sulfuric acid was added to 0.01 g of dry material, and the mixture was incubated at 90°C for 2 hours. Post-incubation, the mixture was centrifuged to remove debris, and 100 µl of the supernatant was diluted with 400 µl of distilled water. To this, 0.5 ml of phenol and 2.5 ml of sulfuric acid were added. The mixture was incubated for 20 minutes at room temperature, followed by 20 minutes at 30°C and another 20 minutes in the dark. Absorbance was measured at 490 nm, and a glucose calibration curve was used for quantification.

• Protein content (%).

Total protein content was quantified using the Fleurence et al. (1995) method (20). Initially, 0.1 g of powdered sample was dissolved in 0.5 ml of distilled water, then heated to 95°C for 1 hour in a Labnet AccuBlock Digital Dry Bath and sonicated at 40 kHz for 90 minutes using a Branson 1510 sonicator bath. Following centrifugation at 12,000 rpm for 20 minutes at 4°C using an Eppendorf 5424 R centrifuge, the supernatant was collected. The pellet was re-suspended in 500 ml of 0.1 M sodium hydroxide and treated similarly, with combined supernatants for protein quantification using the Bradford method (21). Calibration was performed with 98% bovine serum albumin (BSA), and absorbance was measured at 595 nm using an Ultrospec 3100 pro UV/VIS spectrophotometer.

• Lipid content.

Lipids were extracted from plant tissues following the Folch method (22). Plant tissues (0,4g) were homogenized in a solution of water/methanol (99.8%)/chloroform (99–99.4%) at a 1:1:2 (v/v/v) ratio. Samples were placed in an ultrasonic bath (40 kHz, 80 W) for 15 minutes, followed by centrifugation at 4500 rpm at 4 °C for 10 minutes to separate the organic phase. This step was repeated with an additional 4 ml of chloroform. The lipids were then washed with 0.9% NaCl in a separating funnel, after which the solvent was evaporated using nitrogen gas, and the lipids were weighed.

#### 2.2.4 Mineral concentration

Mineral concentration was determined following the method described by Thavarajah et al. (2009). A 500 mg portion of each sample was weighed into a glass tube, followed by the addition of 6 mL of concentrated nitric acid (70%, HNO3). The tubes were placed in a digestion block (QBlock series, Ontario, Canada) and heated at 90 °C for 60 minutes. Subsequently, 3 mL of 30% hydrogen peroxide (H2O2) was added, and the samples were further heated at 90 °C for 15 minutes until most of the residue was digested. Next, 3 mL of 6 M hydrochloric acid (HCl) was added. After cooling to room temperature, the samples were brought to a final volume of 10 mL and filtered. The concentrations of essential minerals, including iron (Fe), zinc (Zn), calcium (Ca), magnesium (Mg), manganese (Mn), sodium (Na), and copper (Cu), were determined using inductively coupled plasma-optical emission spectroscopy (ICP-OES; ICAP-7000 Duo, Thermo Fisher Scientific, France). Calibration curves for each element were established using serial dilutions ranging from 0.1 to 10 mg L^-^¹. Analytical accuracy and precision were validated using internal laboratory standards and certified reference materials from the National Institute of Standards and Technology (NIST).

#### 2.2.5 Nitrogen, phosphorus, and potassium content

The determination of nitrogen (N), phosphorus (P), and potassium (K) was carried out using a Skalar auto-analyzer after acid digestion (Fal et al., 2023). Specifically, 2.5 ml of the digestion solution, composed of sulfuric acid with added salicylic acid and selenium, was combined with 0.4 g of powdered material and allowed to sit for 2 hours. The mixture was then incubated at 100°C for 2 hours. After cooling, 1 ml of 30% hydrogen peroxide was added in three separate portions, allowing sufficient time for the reaction to proceed fully. A final incubation was performed at 330°C for 2 hours. The digested product was then diluted with distilled water and analyzed using the Skalar Scan++ system at the Plant and Microbial Biotechnology Center, MAScIR Foundation, Mohammed VI Polytechnic University.

#### 2.2.6 Amino acid composition

Amino acid analysis was performed as described by (23) using a SHIMADZU (Japan) liquid chromatography system coupled with an MS 8050 mass spectrometer. A 40 mg aliquot of plant extract was hydrolyzed with 10 mL of 6 M HCl at 110°C for 22 hours. Following hydrolysis, the sample was cooled to 4°C to terminate the reaction and subsequently diluted to 50 mL with distilled water. The pH of the hydrolysate was adjusted to 4.5 and filtered through a 0.22 µm PTFE membrane to remove particulates.

Chromatographic separation was conducted at 40°C using a Shim-pack GIST PFPP column (2.1 mm I.D. × 150 mm, 3.0 µm; Kyoto, Japan). A gradient elution was employed with water (solvent A) and acetonitrile (solvent B), both containing 0.1% formic acid, at a flow rate of 0.25 mL/min. The injection volume was 3 µL. The gradient program was as follows: 0–2 min, 100% A; 5 min, 75% A; 11 min, 65% A; 16 min, 50% A; 19 min, 5% A; followed by 100% B from 30 to 32 min, and a 4 min hold.

Mass spectrometric detection was carried out in both positive and negative electrospray ionization (ESI) modes.

#### 2.2.7 Antinutritional compounds

• **Total phenolics content (TPC)**.

Total phenolic compounds were quantified using the Folin–Ciocalteu colorimetric method. The assay is based on the reduction of the Folin–Ciocalteu reagent by hydroxyl groups present in the sample. In a 96-well microplate, 20 µL of each extract was mixed with 100 µL of 10-fold diluted Folin–Ciocalteu reagent and 80 µL of 7% sodium carbonate (Na_2_CO_3_). The reaction mixture was incubated at room temperature in the dark for 30 minutes, and absorbance was measured at 725 nm. Gallic acid was used to generate the standard calibration curve. Results were expressed as milligrams of gallic acid equivalent per gram of extract (mg GAE/g extract).

• Total flavonoids content (TFC).

Flavonoid content was determined based on the formation of a stable complex between aluminum chloride and the flavonoid skeleton. Briefly, 100 µL of sample extract was mixed with 50 µL of 1.2% aluminum chloride and 50 µL of 120 mM potassium acetate. The mixture was incubated in the dark for 30 minutes, and absorbance was recorded at 415 nm. A quercetin standard curve was prepared under identical conditions. The results were expressed in milligrams of quercetin equivalent per gram of extract (mg QE/g extract).

• Vicine (VC) and Convicine (CC) contents.

Vicine and convicine characterization and quantification were analyzed using a Shimadzu Japan system coupled to an MS 8050 mass spectrometer. A triple extraction method to extract vicine and convicine from faba bean flour as described by Pulkkinen et al. (24). 17. 25 mg of flour was weighed and subjected to three successive extractions using 1 mL of 7% perchloric acid (HClO_4_) per extraction. Each extraction involved vortexing, followed by 10 minutes of sonication, after the samples were centrifuged at 6,000 × g for 10 minutes at room temperature. The supernatants were collected after each extraction and pooled together, yielding a total extract volume of 3 mL. The pH of the extract was adjusted to 1.5 by the addition of 1 mL of 9% sodium hydroxide (NaOH) solution, followed by thorough mixing. A final 200-fold dilution (1:200) of the original sample was prepared, samples were stored at -20 °C until further analysis. High-performance liquid chromatography (HPLC) analysis was carried out using a Shimadzu LC-40D system equipped with a reverse-phase C18 column (Zorbax Eclipse XDB-C18, 4.6 × 150 mm, 3.5 µm; Agilent, USA). Separation was achieved using a gradient elution system with water and acetonitrile, each containing 0.1% formic acid, as the mobile phases. The flow rate was set at 1.0 mL/min, and 5 µL of each sample was injected via an autosampler SIL-40C xs. The gradient program starts at 0% B from 0 to 4 min, then shows a gradual increase from 0% to 10% B for 15 min. The compounds are detected in positive mode.

### 2.3 Statistical analysis

The number of evaluated stems, egg laying, adult exit hole, and average of infested faba bean plants, in addition to the total weight and seed weight (kg/ha) and seed size and nutritional quality parameters in each cage, were subjected to square root transformation to normalize variances before analysis. Mean numbers of each parameter were analyzed using one-way analysis of variance (ANOVA) to determine the effects of the treatment (infestation) for each year, followed by the Newman-Keuls test at p ≤ 0.05. The computations were carried out using GenStat (21st Edition, VSN International, UK). Principal Component Analysis (PCA) was performed using the R package FactoMineR, along with a two-way analysis of variance followed by Duncan’s *post hoc* test (p< 0.05), to evaluate the effects of different treatments on seed yield, as well as on phenotypic and nutritional quality traits. In addition, Spearman correlation analysis (p< 0.05) was carried out using the corrplot R package to examine the relationships between measured traits.

## 3 Results

### 3.1 *Lixus algirus* Infestation and impact on seed yield

**Figure 2:**
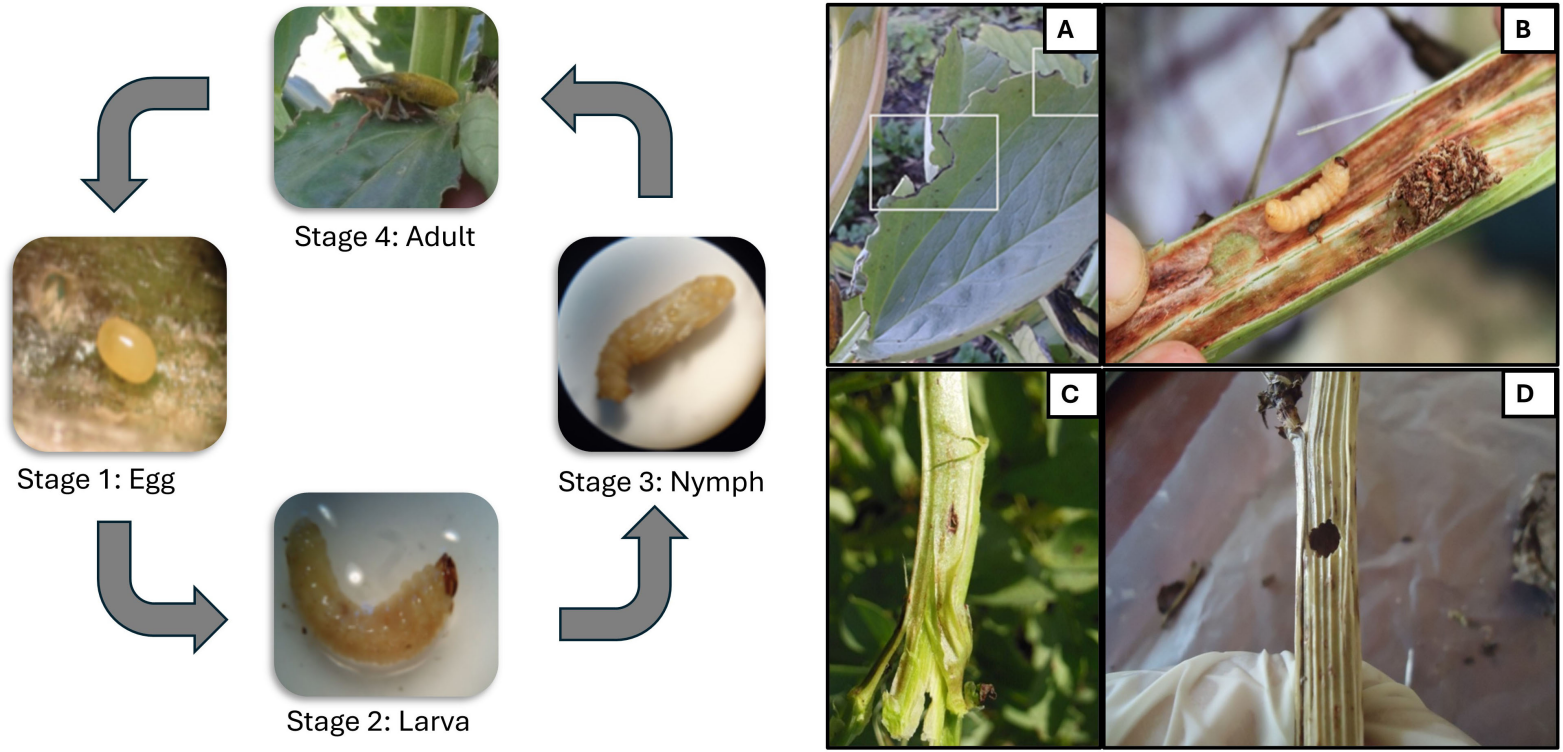
Life cycle and type of damage caused by of both *L. algirus* adult and larva on faba bean plants. The total life cycle (egg to adult) of Lixus algirus lasts approximately 55–60 days under Mediterranean conditions, with larval development inside stems lasting about 42 days (8) Adult feeding results in characteristic marginal, semi-circular notches **(A)**, larvae create feeding tunnels and a dark brown secretion within the stem **(B)**, laying hole **(C)**, adult exit hole **(D)**.

In 2019, 1,279 oviposition holes and 312 adult emergence holes were recorded from 353 assessed stems in infested cages, with L. algirus completing development in 93% of plants. In 2020, fewer oviposition holes (776) and adult exits (180) were recorded from 320 stems, corresponding to 81.7% infestation (**Table 1**). Adults feed on the tender upper leaves from late winter to late spring, creating the characteristic semi-circular notches (**Figure 2**), often accompanied by dark fecal spots. Larval feeding within stems produced brown exudates, disrupted vascular tissues, and caused visible symptoms such as stunting and chlorosis.

**Table 1:** Average infestation rates of faba bean stem borer on 20 faba bean plants in artificial infestation under cages, Marchouch Station, during two cropping seasons (2018–19 and 2019-20).

Year	Total number of evaluated stems/average per plant	Total number of laying holes/average LH	Total number of exit holes/average of EH	% infested plants
2019	353/5.88	1279/21.31	312/5.2	93
2020	320/5.33	776/12.61	180/1.14	81.66

Oviposition began in mid-February, peaked in March, and continued until late April, with activity higher in 2019 (~21 holes per plant) than in 2020 (~13 holes per plant). Larval density increased steadily through April, and pupation peaked in May. Typically, one larva developed per stem, although two larvae were occasionally found, intensifying stem damage. Adult emergence began in mid-May, completing a single annual generation under cage conditions in the local variety ‘Defes’.

Seed and biomass yields were consistently higher in uninfested cages (**Table 2**). Across both seasons, seed yield averaged 4099 kg/ha in controls compared with 3420 kg/ha in infested plants, a reduction of 16.6%. Biomass yield declined similarly, from 7966 kg/ha in controls to 6865 kg/ha under infestation (-13.8%). Yield loss was greatest in 2018/19 (25.3%) and ranged between 14.7% and 20.2% in 2019 and 2020, respectively. These results confirm the substantial negative impact of L. algirus on both reproductive and vegetative performance of faba bean.

**Table 2:** Effect of *L. algirus* infestation on total weight yield, avoidable yield loss, and yield increase over uninfested plants on Moroccan local faba bean variety defes variety at Marchouch station during two cropping seasons: 2018–2019, and 2019–2020.

Treatment	Total biomass yield (kg/ha)		Seed weight (kg/ha)		Seed yield loss (kg/ha)		Avoidable yield loss (%)		Grain yield increase over infested cages (%)	
Years	2019	2020	2019	2020	2019	2020	2019	2020	2019	2020
Infested Cages	6494 a	7235 a	2167 a	4673 a	549	808	20.21	14.74	25.33	17.29
Non-Infested Cages	7228 b	8704 b	2716 b	5481 b						
CV (%)	6.063	10.34	14.68	9.070	–	–	–	–	–	–
SEM	169.8	336.3	146.3	188.0						

Means followed by the same letter(s) within a column do not significantly differ at p = 0.05; CV is the coefficient of variation; SEM is the standard error of the mean.

### 3.2 Impact of L. algirus infestation on seed quality

#### 3.2.1 Seed morphology and cooking time

The impact of L. algirus infestation on faba bean seed morphology and cooking time over two years is shown in (**Table 3**). In 2019, the infested cage seeds had a significantly higher 100-seed weight (HSW) (133.38 g) compared to the control seeds (103.02 g). In 2020, the infested cage seeds also exhibited a higher HSW (123.43 g) than the control seeds (111.34 g), but the difference was not as pronounced as in 2019. The values for HSW in both years were statistically significant.

**Table 3:** Impact of *L. algirus* infestation on the geometry of faba bean seeds and cooking time.

Treatment	Year	HSW (g)	SP (mm ^2^)	SL (mm)	SW (mm)	CT (min)
Infested cage	2019	133.38 ^a^	68.65^a^	19.65 a^,b^	13.72 ^a^	40.82^a^
Non-Infested cages		103.02 ^a^	66.55^b^	19.09 ^a^	13.33^b^	47.40 ^a^
Infested cage	2020	123.43 ^a^	69.95^a^	19.97 a^,b^	14.03^a^	61.16 ^a^
Non-Infested cages		111.34 ^a^	68.74 ^a^	19.72 ^a^	13.71^a^	59.91 ^a^

HSW, Hundred Seed Weight, SP, Seed Perimeter, SL, Seed Length, SW, Seed Width, CT, Cooking Time.

Means followed by the same letter(s) within a column do not significantly differ at p = 0.05.

For the seed perimeter (SP), the infested cage seeds had a larger SP (68.65 mm) compared to the control seeds (66.55 mm) in 2019. In 2020, the infested cage seeds again showed a larger SP (69.95 mm) than the control seeds (68.74 mm), though no significant difference was observed between the two treatments in either year. The values for SP were statistically similar.

Seed length (SL) in 2019 was significantly larger in the infested cage group (19.65 mm) compared to the control group (19.09 mm). In 2020, the infested cage seeds (19.97 mm) had a slightly greater SL than the control seeds (19.72 mm), but no significant difference was found between the two treatments in either year.

Seed width (SW) in 2019 was higher in the infested cage group (13.72 mm) compared to the control group (13.33 mm). In 2020, the infested cage seeds (14.03 mm) had a larger SW than the control seeds (13.71 mm), although no significant difference was found between the treatments, as indicated by the same letter (a). This suggests that L. algirus infestation does not significantly influence SW. Finally, the cooking time (CT) of the seeds varied significantly between the infested and control groups. In 2019, the infested cage seeds required 40.82 minutes to cook, which was significantly shorter than the control seeds (47.40 minutes). In 2020, the infested cage seeds took 61.16 minutes to cook, which was significantly longer than the seeds control (59.91 minutes).

#### 3.2.2 Seed nutritional quality

Faba bean infestation by L. algirus had a significant impact on the nutritional composition of faba bean seeds, with notable increases in protein, carbohydrate, and starch content (**Table 4**). In 2019, infestation led to a 23.48% increase in protein content (from 22.15% to 27.35%) and a 30.92% increase in carbohydrate content (from 1.52% to 1.99%) compared to uninfested seeds. Fiber content remained relatively stable with only a 1.07% increase (from 30.80% to 30.47%). Ash content showed a 14.92% increase (from 2.48% to 2.85%), whereas lipid content decreased slightly by 25.00% (from 0.004% to 0.003%).

**Table 4:** Proximate composition of faba bean seeds from infested and uninfested faba bean plants, under cages.

Treatment	Year	MC	PC	CH	LC	FC	AC	SC
Infested cages	2019	7.74 ^a^	27.35^a^	1.99 ^a^	0.003 ^a^	30.47 ^a^	2.85 ^a^	5.08 ^a^
Non-Infested cages		7.74 ^a^	22.15 ^a^	1.52 ^a^	0.004 ^a^	30.80 ^a^	2.48 ^a^	5.17 ^a^
Infested cages	2020	8.07 ^a^	25.08 ^a^	2.12 ^a^	0.005 ^a^	29.94 ^a^	2.97 ^a^	4.51 ^a^
Non-Infested cages		8.61 ^a^	22.17 ^a^	0.94 ^a^	0.004 ^a^	33.86 ^a^	3.47 ^a^	5.27 ^a^

MC, Moisture content, PC, Protein content, CH, Carbohydrate content, LC, Lipid content, FC, Fiber content, AC , Ash content, SC , Starch content.

Means followed by the same letter(s) within a column do not significantly differ at p = 0.05.

In 2020, similar trends were observed, with protein content increasing by 13.13% (from 22.17% to 25.08%) and carbohydrate content experiencing a drastic 125.53% increase (from 0.94% to 2.12%), indicating substantial enrichment. Starch content also increased by 14.42% (from 5.27% to 4.51%). However, fiber and ash content decreased by 11.58% (from 33.86% to 29.94%) and 14.41% (from 3.47% to 2.97%), respectively, suggesting possible structural depletion in response to infestation. Lipid content showed a 25.00% increase (from 0.004% to 0.005%) compared to the control.

##### 3.2.2.1 Impact L. algirus infestation on mineral composition

The combined results over the 2019 and 2020 seasons revealed that L. algirus infestation significantly compromised the mineral composition of faba bean seeds (**Table 5**). Seeds from infested plants showed marked reductions in several key micronutrients, including zinc (-30.47%), iron (-27.57%), and manganese (-44.61%). Similar declines were observed for copper (-41.68%), magnesium (-41.75%), calcium (-42.03%), sodium (-39.79%), and boron (-22.34%), all of which were significantly lower in infested treatments compared to non-infested controls.

**Table 5:** Impact of L. algirus infestation on faba bean seed mineral composition.

Element	Non infested	Infested	Decrease (%)
Zn (mg/kg)	39.55 ± 2.79 a	27.50 ± 1.71 b	30.47
Fe (mg/kg)	79.07 ± 4.26 a	57.27 ± 3.74 b	27.57
Mn (mg/kg)	429.12 ± 23.02 a	237.71 ± 32.00 b	44.61
Cu (mg/kg)	89.06 ± 4.09 a	51.94 ± 11.13 b	41.68
Mg (mg/kg)	195.72 ± 27.66 a	114.01 ± 23.16 b	41.75
Ca (mg/kg)	489.22 ± 9.41 a	283.60 ± 58.49 b	42.03
Na (mg/kg)	6321.31 ± 849.43 a	3806.10 ± 701.84 b	39.79
Ni (mg/kg)	3.32 ± 0.14 a	2.76 ± 0.23 a	16.87
B (mg/kg)	9.85 ± 0.42 a	7.65 ± 0.26 b	22.34
N (%)	4.15 ± 0.19 a	4.11 ± 0.15 a	0.96
P (%)	0.59 ± 0.03 a	0.58 ± 0.02 a	1.69
K (%)	1.25 ± 0.02 a	1.28 ± 0.02 a	-2.4

Means followed by the same letter(s) within a row do not significantly differ at p = 0.05.

Although a moderate decrease in nickel content (-16.87%) was noted, it was not statistically significant. Furthermore, the macronutrients nitrogen (N), phosphorus (P), and potassium (K) remained statistically unchanged between treatments, suggesting a degree of physiological resilience or homeostatic regulation in their accumulation, even under biotic stress. These findings indicate that L. algirus infestation specifically impairs micronutrient deposition, potentially disrupting the plant’s nutrient uptake or translocation mechanisms.

##### 3.2.2.2 Impact L. algirus infestation on amino acid

The infestation by L. algirus was associated with significant alterations in the amino acid composition of faba bean seeds, with several compounds showing elevated concentrations in infested plants (**Table 6**). Aspartic acid increased from 23.48 mg/kg in non-infested seeds to 26.06 mg/kg under infestation. Glutamic acid rose from 82.46 mg/kg to 88.32 mg/kg, and threonine from 30.79 mg/kg to 32.60 mg/kg. The most substantial increases were observed for leucine and arginine, which reached 8182.27 mg/kg and 1516.78 mg/kg in infested samples, compared to 6439.14 mg/kg and 1158.09 mg/kg in controls, respectively. Among these, only the increase in arginine was statistically significant (p< 0.05).

**Table 6:** Average amino acid concentrations (mg/kg) in seeds from faba bean plants infested and non-infested by L. algirus across two cropping seasons (2019 and 2020).

Amino acids	Amino acid content (mg/Kg)	
	Infested	Non infested
Aspartic acid	26.06^a^	23.48^a^
Hydroxyprolin	10.09^a^	10.02^a^
Glutamic acid	88.32^a^	82.46^a^
Threonin	32.6^a^	30.79^a^
Alanin	50.23^a^	47.26^a^
Valin	24.73^a^	28.85^a^
Glutamin	197.41^a^	184.76^a^
Lysin	188.39^a^	179^a^
Histidin	86.41^a^	83.12^a^
Leucin	8182.27^a^	6439.14^a^
Arginin	1516.78^a^	1158.09^b^
Tyrosin	365.17^a^	314.63^a^
Isoleucin	7676.91^a^	6586.56^a^
Phenylalanin	2558.23^a^	2660.37^a^

Means followed by the same letter(s) within a column do not significantly differ at p = 0.05.

For most other amino acids, including glutamine, lysine, alanine, isoleucine, tyrosine, and phenylalanine, differences between treatments were not statistically significant. Hydroxyproline content remained stable across treatments. These results suggest that L. Algirus infestation may promote or preserve the biosynthesis of specific amino acids, potentially as part of a physiological adaptation to biotic stress.

##### 3.2.2.3 Impact of L. algirus infestation on antinutrients

Total phenolic content ranged from 75.84 to 87.36 mg/ml, with the highest value observed in infested samples in 2019 (87.36 mg/ml), while the other conditions showed slightly lower but comparable values (**Table 7**). TFC was highest under infestation in 2019 (3.10 mg/ml) but declined in 2020 (2.67 mg/ml), whereas non-infested samples exhibited more stable values (2.21-2.27 mg/ml). Vicin (VC) levels varied across treatments and years, reaching the highest concentration in infested samples in 2019 (10.71 mg/g) and the lowest in non-infested samples in the same year (9.42 mg/g). Convicin (CC) followed a similar trend, with slightly higher levels in non-infested conditions, peaking at 3.85 mg/g in 2020 and decreasing to 2.92 mg/g in 2019, while infested samples ranged from 3.55 to 3.57 mg/g.

**Table 7:** Impact of L. algirus infestation on faba bean antinutrients.

Treatment	Infested			Non infested		
Year	2019	2020	Average	2019	2020	Average
TPC (mg/ml)	87.36^a^	76.56^a^	81.96	75.84^a^	79.13^a^	77.48
TFC (mg/ml)	3.10^a^	2.67^a^	2.88	2.21^a^	2.27^a^	2.24
VC (mg/g)	10.71^a^	10.18^a^	10.44	9.42^a^	10.37^a^	9.89
CC (mg/g)	3.55^a^	3.57^a^	3.56	2.92^a^	3.85^b^	3.38

Means followed by the same letter(s) within a column do not significantly differ at p = 0.05.

### 3.3 Multivariate analysis of nutrient and trait variability

#### 3.3.1 Correlations

Under infested conditions, several strong positive correlations were observed among mineral traits, with zinc and iron showing the most consistent association (r = 0.99, p< 0.001), followed by close relationships among calcium, sodium, magnesium, manganese, and copper. Seed traits also interacted with nutritional parameters, as seed weight was positively linked to sodium, while zinc tended to associate with cooking time. Seed yield showed a positive association with lipid content.

Among biochemical traits, glutamic acid derivatives and threonine were tightly connected, and threonine also correlated with lysine. Aspartic acid was positively associated with potassium, and seed perimeter correlated positively with protein content but negatively with lipid content.

Under non-infested conditions, similar trends emerged. Iron and zinc again displayed a strong correlation (r = 0.98), as did calcium, magnesium, and sodium. Manganese was associated with both calcium and magnesium, while copper correlated with iron. Lipid content was positively linked with seed weight, whereas zinc was inversely related (r = - 0.86). In this context, seed yield was negatively associated with cooking time. For amino acid profiles, threonine, lysine, and glutamic acid were closely interconnected with seed yield, although glutamic acid also showed some unexpected negative associations with aspartic acid and yield.

#### 3.3.2 Principal component analysis

**Figure 3:**
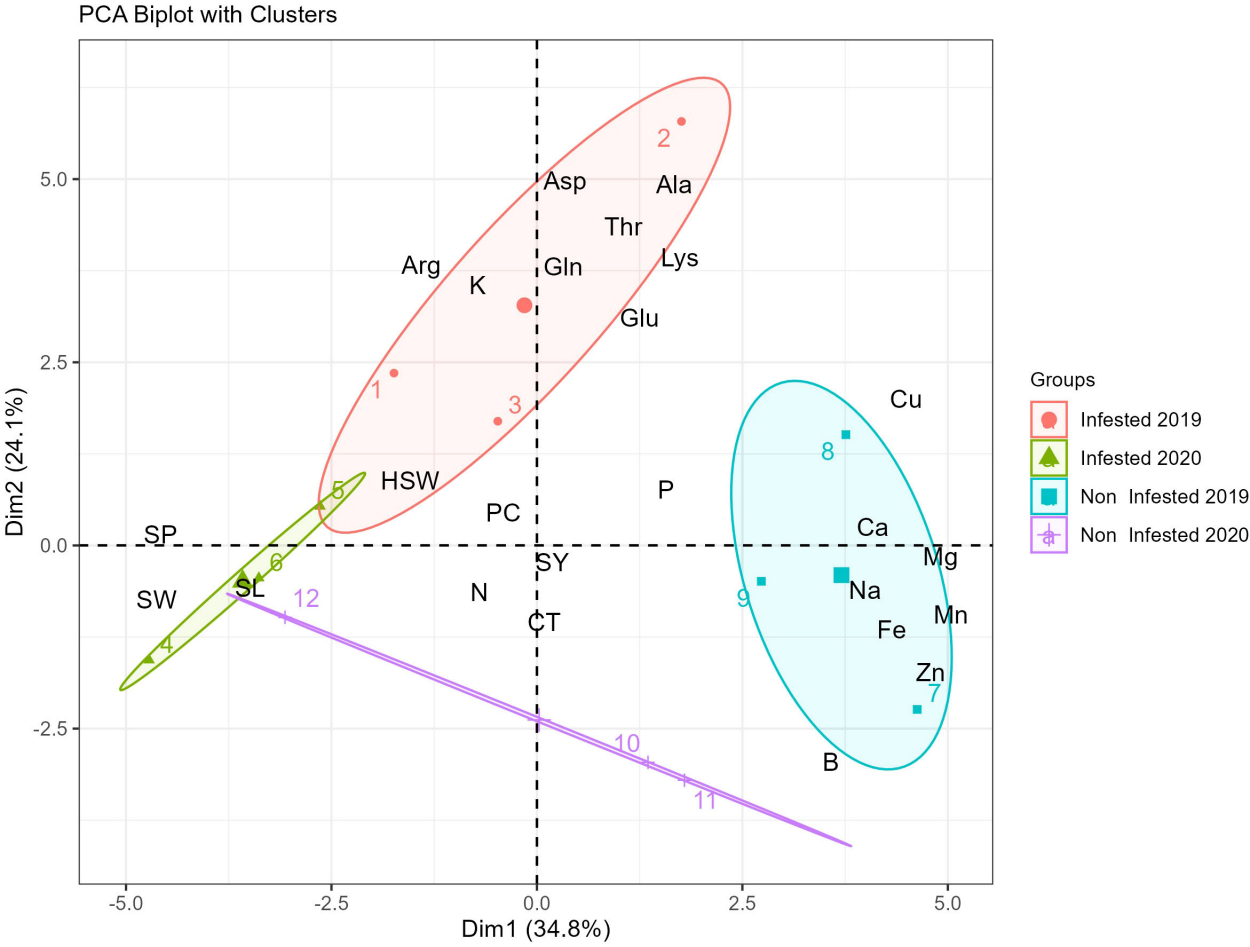
PCA biplot of faba bean samples showing separation by infestation status and year based on nutrient composition and seed traits.

The PCA biplot provides valuable insights into the variation in nutrient composition and seed morphological traits of faba beans under infested and non-infested conditions across different years (**Figure 3**). The first two principal components (Dim1: 34.8% and Dim2: 24.1%) collectively explained 58.9% of the total variance, indicating that these components capture a significant portion of the dataset’s variability. The clustering patterns observed in the biplot revealed distinct groupings between infested and non-infested samples from both 2019 and 2020, highlighting key nutrient and morphological differences between these conditions.

Infested samples from 2019 (represented in red) showed a strong association with increased levels of amino acids such as aspartic acid (Asp), alanine (Ala), threonine (Thr), lysine (Lys), and glutamic acid (Glu). Conversely, non-infested samples from 2019 (represented in blue) are closely linked to higher concentrations of essential minerals, including zinc (Zn), iron (Fe), sodium (Na), manganese (Mn), and calcium (Ca).

Infested samples from 2020 (represented in green) exhibited a distinct pattern, being primarily associated with seed morphological traits such as seed perimeter (SP), seed width (SW), and seed length (SL). Furthermore, the PCA biplot reveals that certain variables, such as seed yield (SY), cooking time (CT), and nitrogen (N), are positioned centrally, suggesting that these traits exert a more balanced influence across all sample groups.

## 4 Discussion

### 4.1 Damage and infestation dynamics

According to the current study, the infestation of the faba bean stem borer led to a decrease in the overall seed weight under cages, ranging from 14.74 to 20.21%. The present findings are not consistent with those conducted in Syria in 1984/85 and 1985/86 seasons that show less yield loss that is estimated at between 3.78 to 13.90% reduction of grain yield of faba bean, artificially infested with a level two times as much as the average natural populations (1.5 females/m2), using Syrian local Medium faba bean (17).

The current study showed the maximum faba bean stem borer damage in 2019 with up to 1279 laying holes and 312 adult exit holes, representing 93% of infested plants, which reflected the highest grain loss of about 20.21% compared to the control. In the second year, 81.66% of infested plants were recorded, with 776 laying holes and 180 exit holes, resulting in less grain yield loss, with about 14.74%. These differences in terms of level of infestation and grain yield loss in different years could be related to environmental conditions. Similarly, increased L. algirus infestation was found in faba bean fields in some coastal regions of Syria, Lebanon, and Turkey that experience high levels of precipitation and humidity (25). According to (17) Cardona et al, the economic significance of L. algirus appears to differ by region. According to (8)Ait Taadaouit et al. (2021), the development and trend of population fluctuations of L. algirus on different faba bean varieties were influenced by seasonal differences in meteorological conditions in Morocco.

A similar observation was reported by Ait Taadaouit et al. (2021a, 2021b) (8, 15) showed almost the same life cycle of the insect under field conditions in Marchouch and Douyet regions during three cropping seasons from 2015 to 2017. However, more egg laying and exit holes were pronounced under artificial infestation under cages and under higher pressure of L. algirus, where the new generation of adults emerges a few days earlier compared to the field conditions.

Throughout its area of distribution, Morocco, Tunisia, Syria, Italy, and Spain, a similar damage has been observed for this pest (8, 10, 12, 17).

### 4.2 Effects on seed morphology

Despite significant yield losses caused by L. algirus infestation, seed morphology remained relatively stable with a slight non-significant increase in perimeter, length, width, and primarily for seed area for infested plants compared to the control. Some individuals of L. algirus damage the flowers, leading to reduced seed set. While the seeds that develop might not be smaller, the overall number of seeds produced per plant is lower. Previous research showed that moderate drought stress had a negative effect on the pod number per plant but had no effect on the seed size or seed number per pod (26).

In cowpea (Vigna unguiculata), Maruca vitrata infestation significantly reduces pod numbers per plant, yet seed traits such as 100-seed weight and size remain unaffected. This stability suggests a resource reallocation mechanism, where plants prioritize seed development over pod production to ensure reproductive success under stress (27). Similarly, in greengram (Vigna radiata), drought stress reduces pod count without impacting seed geometry or weight, indicating a conserved strategy to maintain seed quality despite environmental challenges (28).

Our findings align with these results, demonstrating that seed physical measurements, including 100-seed weight and size, remain consistent under biotic and abiotic stress. While pod numbers were not assessed in our study, the observed preservation of seed characteristics supports the hypothesis that plants allocate resources to protect seed quality under adverse conditions. This resilience underscores the importance of prioritizing seed traits in breeding programs aimed at improving stress tolerance.

### 4.3 Biochemical and nutritional alterations

In contrast to the stability of morphological traits, seed biochemical composition was markedly altered by infestation The mean protein concentration in faba bean seeds increased significantly in infested plants compared to healthy ones, with values of 23.52% and 13.12% recorded in 2019 and 2020, respectively. This trend aligns with findings in other plant species, where biotic and abiotic stresses often lead to alterations in seed protein content and composition, and field insect pest infestation frequently leads to increased seed protein levels alongside changes in amino-acid profiles across major crops (29).

For instance, a study conducted in Mississippi, USA, reported a significant increase in protein concentration in soybean (Glycine max) seeds infected with Cercospora kikuchii, the causative agent of purple seed stain (PSS). The protein content rose from 36.8% in healthy seeds to 37.8% in seeds from infested plants, along with notable changes in amino acid profiles, suggesting that infection by C. kikuchii not only increases total protein levels but also influences protein composition (30).

Similarly, in narrow-leafed lupin (Lupinus angustifolius), exposure to fungal pathogens such as Colletotrichum lupini, which causes anthracnose, has been shown to induce the accumulation of specific seed storage proteins, including β-conglutins. These proteins exhibit antifungal properties, highlighting their dual role in plant defense and storage. Although the quantitative increase in total protein content due to pathogen exposure was not explicitly reported, the induced protein synthesis underscores the potential for stress-related protein augmentation (31).

The amino acids composition of the infested faba bean seeds from were increased compared to seeds from uninfested plants. This response contrasts with findings from a study on soybean seeds infected with Cercospora kikuchii, which reported a marked reduction in most amino acids, including lysine, glutamic acid, glycine, valine, methionine, and leucine, with only arginine levels increasing. The increase in amino acids observed in faba beans suggests a more active reallocation of nitrogen toward defense-related metabolic pathways, potentially supporting the synthesis of proteins and secondary metabolites necessary for stress resilience. The contrasting responses highlight species-specific differences in amino acid dynamics under biotic stress, underscoring the diverse ways plants adapt to pest or pathogen challenges (31). In the study by Shikta et al. (2018) (32), the variations in amino acid profiles under biotic stress were linked to specific insect infestations for mustard and sunflower infested by aphids (Lipaphis erysimi)., which exhibited an increase in amino acid levels. Aphid infestation triggered a metabolic reallocation in these oilseeds to enhance stress adaptation, including the synthesis of amino acid-derived defense compounds.

These findings contrast with our results in L. algirus infested faba bean seeds, where a consistent increase in amino acid content was observed. This suggests that faba beans, like mustard and sunflower, exhibit a metabolic response characterized by increased amino acid production to support defense-related pathways. The variation in responses among crops and pests underscores the complexity of plant metabolic adjustments to biotic stress and highlights the role of pest type in influencing these biochemical changes.

For mineral composition, our study demonstrates that L. algirus infestation significantly impacts the mineral content in faba bean seeds. The feeding activity of stem-boring larvae disrupts nutrient transport within the plant, impairing the movement of essential minerals such as calcium (Ca), zinc (Zn), iron (Fe), and magnesium (Mg) to the developing seeds. Since no studies have directly investigated the impact of biotic stressors like insect infestation on the nutritional quality of legume seeds, we drew comparisons from studies examining the effects of abiotic stresses such as drought and heat, which similarly disrupt nutrient dynamics.

For instance, heat stress in lentils caused reductions of 59% in zinc, 52% in iron, 35% in magnesium, and 28% in calcium, with more pronounced effects observed in heat-sensitive genotypes compared to heat-tolerant ones (33). Similarly, chickpea seeds subjected to drought and heat stress exhibited a 10–39% reduction in iron and a 10–31% reduction in zinc content (34). These significant losses were attributed to disrupted nutrient uptake and reallocation toward stress defense mechanisms. In mung bean, salt stress caused notable declines in magnesium and manganese levels, alongside reductions in total carbohydrates and free amino acids (35).

The biotic stress imposed by L. algirus likely triggers similar physiological disruptions, as larvae create feeding tunnels within faba bean stems, obstructing vascular tissues and limiting nutrient transport to pods and seeds. This not only diminishes mineral content but also weakens the plant’s ability to maintain adequate growth and seed quality. Moreover, the infestation-induced stress could stimulate a reallocation of resources toward secondary metabolite production and defense responses, further exacerbating mineral deficiencies in seeds.

While reductions in calcium and zinc were the most significant under abiotic stresses in lentils and chickpeas (33), faba bean seeds under L. algirus infestation show a comparable pattern, as these minerals are critical for seed development and overall plant metabolism. Calcium plays a key role in cellular structure and signaling, while zinc is essential for enzymatic activity and protein synthesis. The depletion of these minerals under biotic stress highlights the detrimental effect of infestation on seed nutritional quality.

### 4.4 Multivariate analysis of nutrient and trait variability

**Figure 4:**
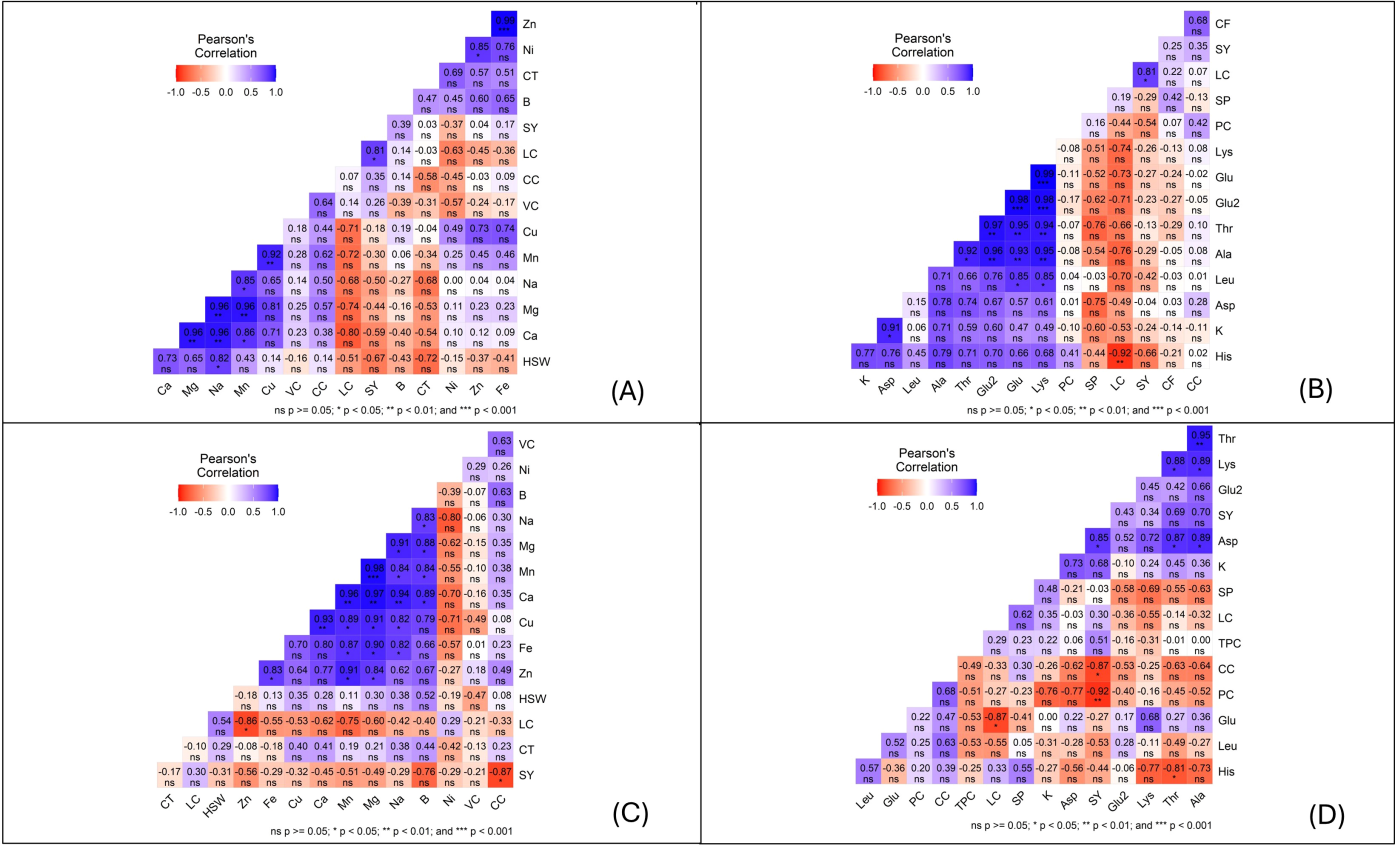
Pearson correlation heatmaps showing the relationships among mineral, biochemical, and morphological traits of faba bean seeds under different conditions. Panels: **(A)** Infested Set 1, **(B)** Infested Set 2, **(C)** Non-infested Set 1 and **(D)** Non-infested Set 2. The color scale represents the strength and direction of correlations (blue = positive, red = negative), with significance levels indicated as p< 0.05 (), p< 0.01 (), and p< 0.001 (); ns = not significant.

The correlation analysis revealed key nutrient interactions influencing plant health under infestation. A strong positive correlation between zinc (Zn) and iron (Fe) (**Figure 4**) suggests a shared metabolic role and co-regulation under stress, consistent with previous findings (36).

Similarly, calcium (Ca), sodium (Na), and magnesium (Mg) showed significant intercorrelations, supporting their role in maintaining ionic and physiological balance under stress (37).

The Mn–Cu correlation highlights their combined function in enzymatic defenses like lignin biosynthesis and oxidative stress regulation (38). Mn’s correlation with Na may point to shared roles in osmotic regulation and stress adaptation, despite limited direct documentation of their interaction (39).

Physiological traits also showed relevant patterns. Lipid content (LC) positively correlated with hundred-seed weight (HSW), while Zn showed a negative correlation with HSW in non-infested samples. Additionally, seed yield (SY) negatively correlated with cooking time (CT), suggesting that higher yields may reduce (CT) due to altered seed composition (36).

Overall, these findings underscore the critical role of nutrient interactions in both infested and non-infested faba beans. In infested plants, certain elements appear to work together to mitigate stress effects or facilitate physiological adaptations, potentially enhancing plant resilience. This suggests that targeted nutrient management strategies could improve plant vigor and reduce susceptibility to damage. Meanwhile, in non-infested conditions, these correlations provide insights into how nutrient balance contributes to plant growth and productivity. Together, these findings highlight the importance of optimizing nutrient availability to support plant health, whether under stress or optimal growing conditions.

The Principal Component Analysis biplot revealed clear distinctions in nutrient composition and seed morphological traits between infested and non-infested samples across years (**Figure 3**). In 2019, infested plants were associated with elevated amino acids (Asp, Ala, Thr, Lys, Glu), suggesting stress-induced metabolic adjustments, while controls clustered with higher mineral concentrations. These patterns indicate that infestation influences both biochemical and physical seed attributes as part of adaptive responses to pest pressure. Similar responses have been reported under biotic and abiotic stress, including changes in amino acid profiles following Botrytis fabae infection (40) Under drought stress conditions, elevated levels of Asp have been observed in several plant species, suggesting its involvement in stress acclimation processes (41).

Glutamic acid also plays a central role in defense-related metabolism and may mediate resistance or susceptibility depending on the stress context (42). In addition, environmental conditions during cultivation have a significant impact on the faba bean amino acid profile, which may also explain these observed changes (43). Conversely, higher concentrations of zinc (Zn), iron (Fe), sodium (Na), manganese (Mn), and calcium (Ca) in non-infested faba bean samples from 2019 may contribute to structural integrity and physiological function, thereby enhancing resistance to pests and diseases. In 2020, infested samples showed stronger correlations with seed morphological traits (seed perimeter, width, and length), suggesting that pest impact extended beyond chemical composition to physical seed development (44). Meanwhile, traits like SY, CT, and nitrogen (N) were centrally positioned in the PCA biplot, indicating consistent influence across conditions. This suggests that, despite stress-induced variation in biochemical and structural traits, core seed qualities like yield and cooking performance remain stable.

### 4.5 Future perspectives

This study highlights the need for further research on cross-crop comparisons to gain a better understanding of borer pest impacts; developing germplasm combining stem borer resistance with enhanced nutritional quality, with a focus on mineral content, and molecular approaches, including genomics, transcriptomics, proteomics, or metabolomics to unravel the mechanisms of stress response and guide breeding programs for resilient, nutritionally secure varieties of faba bean.

## 5 Conclusion

This study demonstrates that Lixus algirus infestation significantly reduces faba bean yield and alters seed nutritional quality. Despite severe stem and leaf damage, seed physical traits remained largely stable, suggesting resource reallocation to preserve seed morphology. Infestation increased protein and amino acid contents but caused sharp reductions in essential minerals such as magnesium, iron, calcium, and zinc, revealing a novel trade-off between nutrient enrichment and depletion. Anti-nutritional compounds were mostly unaffected, although convicine levels varied between years. Multivariate analysis further confirmed distinct differences between infested and non-infested plants, highlighting the complex impact of L. algirus on plant physiology and grain quality. These findings provide the first evidence that borer insect infestation can drive nutritional shifts in legumes, underscoring the need for future research on breeding resistant, nutritionally robust varieties and effective pest management strategies.

## Data Availability

The original contributions presented in the study are included in the article/supplementary material. Further inquiries can be directed to the corresponding author.
